# Aquaporin‐4 in Narcolepsy Type 1: Investigation of Perivascular Fluid Movement in Sleep Disorders

**DOI:** 10.1002/acn3.70398

**Published:** 2026-04-14

**Authors:** Jonas Ranke, Petra Steinacker, Steffen Halbgebauer, Patrick Oeckl, Geert Mayer, Markus Otto

**Affiliations:** ^1^ Department of Neurology Martin‐Luther‐University Halle‐Wittenberg Halle (Saale) Germany; ^2^ Department of Neurology Ulm University Hospital Ulm Germany; ^3^ German Center for Neurodegenerative Diseases (DZNE e.V.) Ulm Germany; ^4^ Department of Neurology Hephata Klinik Schwalmstadt‐Treysa Germany

**Keywords:** Aquaporin‐4, biomarker, glymphatic system, narcolepsy type 1, sleep disorders

## Abstract

Narcolepsy type 1 (NT1) is caused by the loss of hypocretin‐1 leading to excessive daytime sleepiness and cataplexy. Additionally, disrupted nighttime sleep has become an increasingly recognized feature of NT1. As the glymphatic fluid movement has been linked to sleep architecture, we investigated cerebrospinal fluid (CSF) Aquaporin‐4 (AQP4) as potential biomarker for perivascular fluid exchange processes in 133 NT1 patients, 145 patients with other sleeping disorders, and 62 controls. NT1 patients showed lower CSF AQP4 concentrations compared to both control groups (*p* < 0.0001 and *p* = 0.01). AQP4 levels were correlated to the CSF hypocretin‐1 levels. These data suggest potential brain fluid barrier alterations in NT1.

## 1 Introduction

Narcolepsy type 1 (NT1) is a rare neuroimmunological sleeping disorder [1]. The disease is characterized by an autoimmune‐mediated destruction of hypocretin‐1 (hcrt‐1) producing neurons in the hypothalamus [2] leading to the cardinal symptoms of excessive daytime sleepiness and cataplexy. Decreased cerebrospinal fluid (CSF) hcrt‐1 levels are currently the only available fluid biomarker for the diagnosis of NT1 apart from clinical and polysomnographic findings [3]. Consequently, there is a lack of easily available diagnostic, prognostic, and therapeutic biomarkers for NT1.

According to earlier studies [1, 4] up to 60% of NT1 patients additionally suffer from disrupted nighttime sleep (DNS). Corresponding to that, a meta‐analysis found significantly higher shares of early sleep stages (N1) and a higher arousal index in the polysomnography of NT1 patients [5].

An intact sleep architecture, especially sufficient shares of slow wave sleep, is required for the consolidation of memory in the hippocampus [6] Furthermore, sleep is discussed as an important modulator of the glymphatic system [7, 8, 9, 10] which has been perpetually mentioned as a pathophysiological link between the common concomitance of sleeping disorders and cognitive decline [8, 11, 12] Recently, the channel protein aquaporin‐4 (AQP4) which plays a central role in perivascular fluid exchange has been proposed as a potential biomarker of glymphatic dysfunction. AQP4 channels are physiologically expressed on astrocytic endfeet facilitating the exchange of perivascular fluid into the cerebral interstitium and thus mediating perivascular fluid movement. CSF AQP4 has been detectable in several previously mentioned immunoassays under healthy and pathological conditions [13, 14] In line with neuropathological evidence linking dysfunctional polarization of AQP4 on the astrocytes to Alzheimer pathology, several studies have shown significantly increased CSF levels in AD patients compared to healthy controls supporting the diagnostic potential of AQP4 in neurodegenerative disease and glymphatic dysfunction [13, 15, 16].

In this explorative case–control study, we investigated CSF AQP4 levels in NT1 patients compared to patients suffering from other sleeping disorders and controls. We aimed to characterize CSF AQP4 levels to gain further insight into the pathophysiology of NT1 concerning astrocytic protein changes related to perivascular fluid exchange.

## 2 Methods

For this study, CSF of 132 NT1 patients (hcrt‐1 < 110 pg/mL), 143 controls with other sleeping disorders (narcolepsy type 2, idiopathic hypersomnia, secondary hypersomnias) matched for age and sex (hcrt‐1 > 110 pg/mL), and 62 samples from patients without signs of a neurodegenerative or neuroinflammatory disease (headache, dizziness and functional disorders) as a second control group were analyzed. Further demographic information is depicted in Table 1.

**TABLE 1:** Descriptive statistics of the diagnostic cohorts.

	NT1 patients	Other sleep disorders	Further controls
Number of patients	132	143	62
Male	76	74	18
Female	55	69	48
Age at diagnosis Mean ± sd	37.4 ± 8.93 years	37.4 ± 14.59 years	34.8 ± 11.23 years
CSF hypocretin‐1 Median (IQR)	14 pg/mL (10–52 pg/mL)	296 pg/mL (260–340 pg/mL)	n.a.
CSF aquaporin‐4 Median (IQR)	3306 pg/mL (2404–4380 pg/mL)	4148 pg/mL (3232–5199 pg/mL)	3957 pg/mL (3092–4748 pg/mL)

Abbreviations: IQR, interquartile range; NT1, narcolepsy type 1; sd, standard deviation.

The study was conducted according to the revised Declaration of Helsinki and Good Clinical Practice Guidelines. Furthermore, the investigations were approved by the ethics committee of the medical faculty of Martin‐Luther‐University Halle‐Wittenberg, Germany (approval number 2021–101). CSF samples from patients in the cohorts suffering from sleeping disorders were collected during the diagnostic assessment in the neurological departments of cooperating hospitals in Germany. Afterwards, the CSF tubes were kept on dry ice and shipped to our neurological laboratory for the detection of CSF hcrt‐1 (formerly Ulm university hospital, now Halle (Saale) university hospital). The CSF of the validation cohort was sampled in the department of neurology of the university hospital Halle (Saale).

CSF hcrt‐1 levels were determined using a commercially available radioimmunoassay (quantification range 10–1280 pg/mL, Phoenix pharmaceuticals Inc., California, USA). To measure CSF AQP4 concentrations, we used a new ELISA that has recently been established in our group [16] The lower limit of quantification was determined to be 104 pg/mL. The intra‐assay variability was 4.6% and the inter assay variability was 14%.

The median AQP4 concentrations and interquartile ranges were calculated for each of the diagnostic groups.

Statistical analyses were conducted using GraphPad Prism 10 (GraphPad Software Inc., La Jolla, California, USA) and SPSS. Continuous variables are presented as median and interquartile range (IQR). Group differences in CSF AQP4 concentrations were analyzed using analysis of covariance (ANCOVA) with diagnostic group as fixed factor and age as covariate to correct for age as a confounder. Post hoc pairwise comparisons between diagnostic groups were performed using Bonferroni‐corrected tests derived from the ANCOVA model. Correlations of biomarkers with each other and clinical findings were investigated using Spearman correlation analysis. A *p*‐value of less than 0.05 was considered to be significant.

## 3 Results and Discussion

**FIGURE 1:**
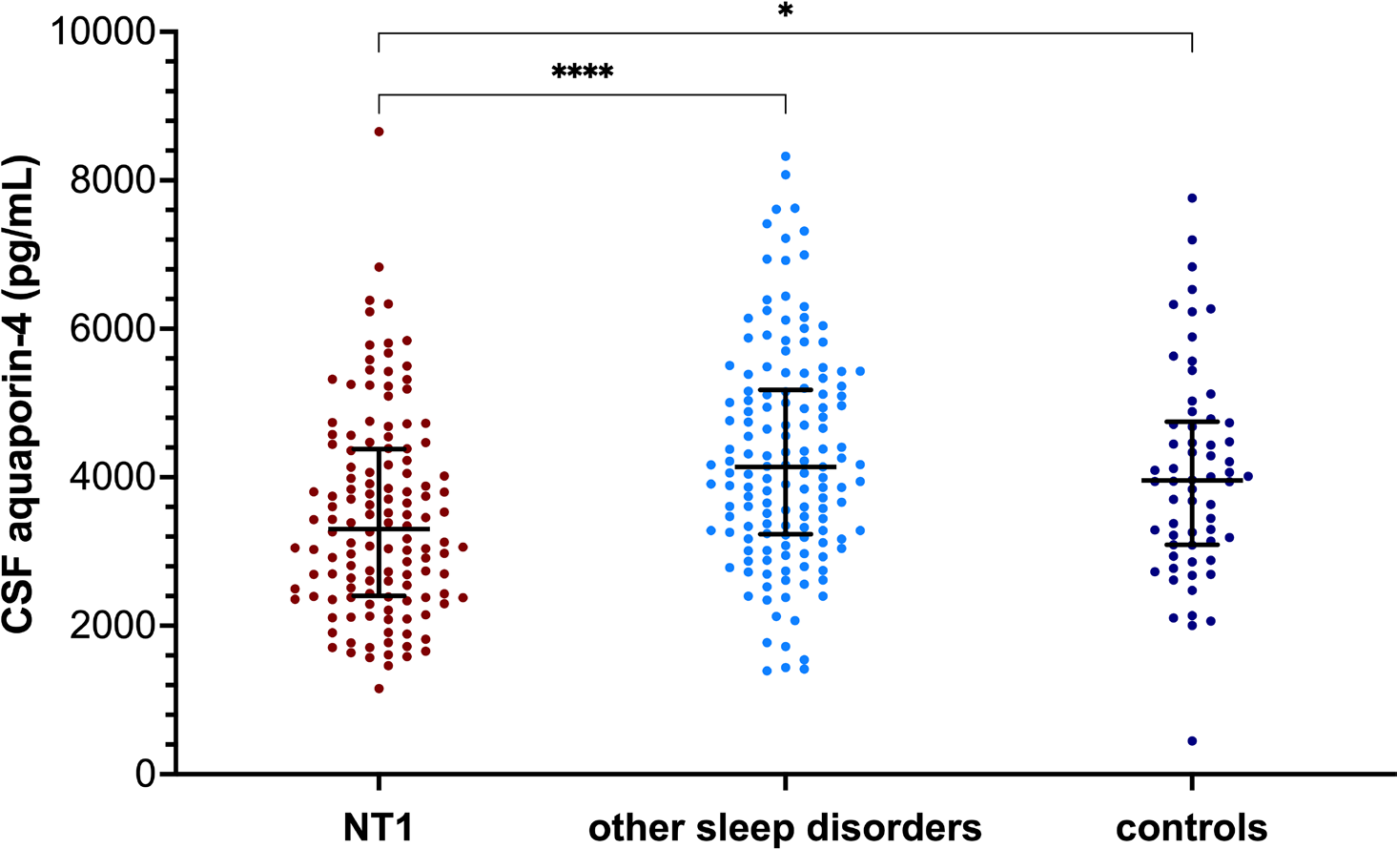
CSF Aquaporin‐4 concentrations of NT1 patients and two control cohorts. Results are depicted as scatter plots showing individual values as well as the median and the interquartile range (IQR) of the CSF aquaporin‐4 concentration of patients with narcolepsy type 1, other sleep disorders (including patients with narcolepsy type 2, idiopathic hypersomnia and secondary hypersomnias) and a second control cohort, respectively. **** *P* < 0.0001. * *P* = 0.01. NT1, narcolepsy type 1.

As illustrated in Figure 1, patients with NT1 showed significantly lower CSF AQP4 concentrations (3306 pg/mL (2404–4380 pg/mL)) than patients of the non‐NT1 sleep disorder group (4148 pg/mL (3232–5199 pg/mL)) as well as patients without sleep disorders (3957 pg/mL (3092–4748 pg/mL); *p* = 0.01). Values are reported as median and interquartile range (IQR).

After adjustment for age as confounder using ANCOVA, the CSF AQP4 concentration differed significantly between diagnostic groups (F (2, 333) =13.31, *p* < 0.001, *η* ^2^ = 0.074). Age was independently associated with AQP4 concentrations (F (1, 333) =26.99, *p* < 0.001, *η* ^2^ = 0.075) in the ANCOVA analysis. We also found a positive, weak correlation of CSF AQP4 concentrations with patient age in the overall cohort in a Spearman correlation analysis (*r* = 0.20; *p* = 0.0002). Post hoc pairwise comparisons confirmed significantly lower AQP4 levels in NT1 patients compared to both age‐ and sex‐matched control groups, whereas no difference was observed between the two control cohorts.

**FIGURE 2:**
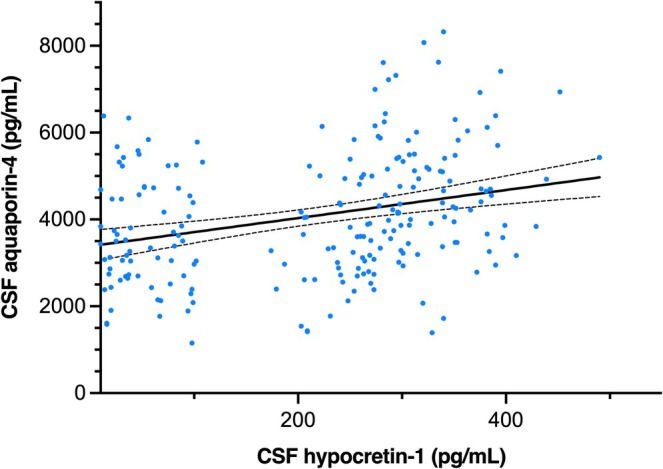
Correlation of CSF Aquaporin‐4 concentration and CSF Hypocretin‐1 levels. This graph shows the Spearman correlation analysis of CSF aquaporin‐4 concentrations and hypocretin‐1 (*n* = 215). Only patients with hcrt‐1 values within the detection limit of the radioimmunoassay were included. Therefore, 60 patients with hcrt‐1 values below 10 pg/mL had to be excluded. Dots are individual values and lines are from linear regression. AQP4 and Hcrt‐1 showed a positive correlation (*r* = 0.31; *p* < 0.0001). Hcrt‐1, hypocretin‐1.

In patients with hypocretin‐1 levels within the quantification range of the commercially available hcrt‐1 radioimmunoassay (*n* = 215), we saw a positive correlation of CSF hcrt‐1 with the detected CSF aquaporin‐4 concentration (*r* = 0.31; *p* < 0.0001, see Figure 2).

To our knowledge, this study represents the first investigation of CSF AQP4 in patients with NT1. Compared to our control cohorts, NT1 patients exhibited significantly lower AQP4 concentrations. In the absence of in vivo or neuropathological data on AQP4 expression in NT1, the biological basis of these reduced levels remains speculative.

AQP4 is predominantly expressed in astrocytic endfeet surrounding cerebral blood vessels at the brain‐fluid interface. Lower CSF biomarker concentrations may therefore reflect altered astrocytic function, reduced astrocytic turnover, or (region‐specific) structural astrocytic changes. Previous studies on in vivo AQP4 expression are scarce, and none have specifically addressed NT1. Of the few neuropathological studies on AQP4 expression in psychiatric disorders, only three have shown reduced expression in conditions like major depressive disorder [17] These studies, however, were post‐mortem and involved patients treated with medications, which could have influenced the findings.

Reduced in vivo expression, however, could explain the lower AQP4 concentrations observed in the CSF of NT1 patients. These mechanisms could be related to the selective hypothalamic pathology of NT1 [2] a brain region known for its high AQP4 expression [18] Regional astrocytic alterations in this area could therefore contribute to lower CSF AQP4 levels in these patients.

The findings in NT1 contrast with reports in neurodegenerative diseases, which have been associated with elevated CSF AQP4 levels. These increases have been interpreted as reflecting astrocytic disruption or altered perivascular fluid dynamics [13, 16] In contrast, lower CSF AQP4 in NT1 may indicate a different astrocytic response pattern. Further studies with larger cohorts are warranted to confirm these findings and explore possible clinical implications of the connection of NT1 pathophysiology with components of the glymphatic system.

The positive correlation of CSF AQP4 and hypocretin‐1 levels with lower levels of both biomarkers in NT1 supports a possible connection of hypocretinergic neurosignalling and perivascular fluid exchange. Hypocretin neurons project densely to noradrenergic brainstem nuclei, particularly the locus coeruleus (LC). Noradrenergic LC neurons have recently been shown to have synchronized activity with arterial pulsation and perivascular fluid movement in vitro models [19] We hypothesize that hypocretinergic projections could modulate astrocytic activity and thus perivascular fluid dynamics via noradrenergic brain stem neurons in NT1 since in vivo imaging studies also found altered brain and vessel pulsation profiles in NT1 [20] Thus, disrupted hypocretinergic‐noradrenergic circuits could be an important mechanism in explaining changed AQP4 concentrations as a biomarker of perivascular fluid transport. Whether alterations in astrocytic AQP4 expression in NT1 and thus CSF AQP4 levels influence perivascular transport processes in vivo remains speculative at the moment and requires further investigation. In line with a previous study from our workgroup, we detected a positive correlation between AQP4 concentration and age [16] Age‐related changes in astrocytic biology and the brain–fluid interface may contribute to altered perivascular transport processes with increasing age, possibly promoting the development of proteinopathies in elderly populations.

A key strength of this study is the large sample size given the low prevalence of NT1. Furthermore, AQP4 was measured using a newly developed non‐commercial ELISA, which provided high analytical sensitivity.

## 4 Conclusion

To conclude, we found significantly lower CSF AQP4 concentrations in a large cohort of NT1 patients compared to both sleep‐disturbed and non‐sleep‐disturbed disease controls. These findings suggest changes in the function of the brain‐fluid‐barrier in NT1, suggesting a potential pathophysiological link between AQP4 as a mediator of perivascular fluid movement and hypocretinergic dysfunction, as reflected by the positive correlation between CSF AQP4 and hypocretin‐1.

## Author Contributions

J.R., P.S., and M.O. contributed to the conception and design of the study; J.R., P.S., G.M., and M.O. contributed to the acquisition and analysis of data; J.R., P.S., S.H., and P.O., G.M., and M.O. contributed to drafting the text, critical revision of the manuscript or preparing the figures.

## Funding

This study was supported by the EU Joint Programme‐Neurodegenerative Diseases networks EU (Moodmarker, PredictFTD, Synapsing), the German Federal Ministry of Education and Research (FTLDc 01GI1007A).

## Conflicts of Interest

J.R. is a participant in the Junior Clinician Scientist Program funded by the Medical Faculty of the Martin Luther University Halle‐Wittenberg. The funding provided J.R. with 20% research time with exemption from clinical obligations.

P.O. received research support from the ALS Association/ALS Finding A Cure (24‐SGP‐691, 23‐PPG‐674‐2), Charcot Foundation (D.7090), Cure Alzheimer's Fund, DZNE Innovation‐to‐Application (I2A_call9_Oeckl), EU Horizon Europe (SYNAPSING), consulting fees from LifeArc and Fundamental Pharma, honoraria for lectures from GSK and travel support from Biogen.

## Data Availability

The data sets that have been gathered and statistically analyzed during the current study are available from the corresponding author on reasonable request and in accordance with institutional and ethical regulations.
